# The effect of roast profiles on the dynamics of titratable acidity during coffee roasting

**DOI:** 10.1038/s41598-024-57256-y

**Published:** 2024-04-08

**Authors:** Laudia Anokye-Bempah, Timothy Styczynski, Natalia de Andrade Teixeira Fernandes, Jacquelyn Gervay-Hague, William D. Ristenpart, Irwin R. Donis-González

**Affiliations:** 1 Department of Biological and Agricultural Engineering, University of California Davis, 3024 Bainer Hall, Davis, CA 95616 USA; 2 Coffee Center, University of California Davis, Davis, CA 95616 USA; 3 Bridge Coffee Co., Marysville, CA 95901 USA; 4 Department of Chemistry, University of California Davis, Davis, CA 95616 USA; 5 Department of Chemical Engineering, University of California Davis, Davis, CA 95616 USA

**Keywords:** Chemistry, Engineering

## Abstract

Coffee professionals have long known that the “roast profile,” i.e., the temperature versus time inside the roaster, strongly affects the flavor and quality of the coffee. A particularly important attribute of brewed coffee is the perceived sourness, which is known to be strongly correlated to the total titratable acidity (TA). Most prior work has focused on laboratory-scale roasters with little control over the roast profile, so the relationship between roast profile in a commercial-scale roaster and the corresponding development of TA to date remains unclear. Here we investigate roast profiles of the same total duration but very different dynamics inside a 5-kg commercial drum roaster, and we show that the TA invariably peaks during first crack and then decays to its original value by second crack. Although the dynamics of the TA development varied with roast profile, the peak TA surprisingly exhibited almost no statistically significant differences among roast profiles. Our results provide insight on how to manipulate and achieve desired sourness during roasting.

## Introduction

The perceived acidity or sourness of brewed coffee is an important sensory attribute that significantly influences coffee quality score, flavor profile, and consumer preference^1–3^. Brewed coffee acidity, which is typically measured through pH and titratable acidity (TA), depends on the quantity and type of acids present in roasted coffee beans, including aliphatic acids like acetic, citric, malic, and quinic acids, as well as a variety of chlorogenic acids^4,5^. Numerous studies have demonstrated a strong correlation between brewed coffee’s perceived sourness and its TA and pH, with TA being the better predictor^2,6–8^.

The TA of coffee can be influenced by several factors, including the green coffee origin^9^, postharvest processing method^10^, roast degree^11^, brewing conditions^2^, and roast profile^12^_._ A roast profile, which is the temperature versus time measured inside the roaster during roasting, is considered one of the most crucial factors, as it controls the chemical reactions in the coffee beans, affecting the flavor, and aroma of brewed coffee^13^. Different roast profiles yield different physical, chemical, and sensory characteristics in roasted coffee beans^14–17^. During roasting, the TA of coffee generally increases up to a peak value, then decreases over time^12^. This change is primarily attributed to the formation of formic and acetic acids from carbohydrates, and the decomposition of chlorogenic acids^12,14,18,19^.

Despite the importance of TA as a correlation for sourness and coffee quality, to date few studies have explored the effect of different roast profiles on TA in coffee during commercial-scale roasting. Instead, several studies have applied indirect observation methods to study the effect of roast profiles on coffee TA while investigating related factors. Ortolá et al.^20^ examined the influence of roasting temperature on the physicochemical properties of different coffees and concluded that higher temperatures yielded higher TA. Wang and Lim^12^ investigated the effect of different roast profiles on the physicochemical properties of roasted coffee and reported that a high-temperature-short-time roast profile (HTST, 240 °C, 3.6 s) resulted in higher TA, when the beans were roasted beyond the beginning of second crack, in comparison to a low-temperature-long-time roast profile (LTLT, 210 °C, 16.9 s). Furthermore, they observed a continuous increase in TA through roasting time, up to a maximum of 5.2 mL NaOH/40 mL sample, followed by its continuous decrease at 48 s after first crack and towards the end of roasting^12^. Similar results were observed by Santos et al.^9^, who reported that TA increased to its maximum at around 200 °C in a fast roast profile (220 °C, 17 min) while increasing over time in a slow roast profile (183 °C, 25 min). Gloess et al.^21^ analyzed the formation of volatile organic compounds during the roasting of different coffees with different roast profiles and observed that the HTST roast profile exhibited higher TA values compared to the LTLT profile. Lastly, Yergenson and Aston^22^ investigated the online determination of coffee roast degree and found that the TA of roasted coffee can be controlled by different isothermal roast profiles, and by using coffee beans from different regions.

The above studies clearly show that roast profile has a significant effect on the development of TA during roasting, but several questions remain unanswered. In particular, most prior studies used isothermal roasting in small-scale roasters with batch sizes ranging from 50 to 250 g, which facilitate isothermal conditions but do not necessarily reflect larger commercial-scale roasting conditions, where the temperature versus time (i.e., the “roast profile”) is a crucial control parameter. Thus, the effects of various dynamic aspects of commercial scale roasting (> 1 kg batch size), including pre-heating, continuous temperature increase, or reduced final temperatures on TA have not been examined. Additionally, previous studies typically collected coffee bean samples only before and after the roasting process, or at infrequent intervals during roasting, providing an incomplete picture of the changes in TA during roasting. Finally, previous studies examined a small set of isothermal roast profiles, such as HTST and LTLT, which do not represent the wide variety of roast profiles that are used in the coffee industry^12^.

Thus, the main goal of this study was to quantify how different roast profiles impact the development of TA in a representative commercial-scale roaster. The study had two main objectives. First, we examined the impact of seven very different roast profiles on the TA of a single-origin coffee, treating the roast profile as the independent factor and the resulting TA as the dependent factor. The total duration of each roast was held constant, but we varied the energy inputs to yield different roast profiles. For instance, some roasts had a high initial heat application followed by a constant decrease in roast energy, while other roasts began with a low initial heat application followed by a gradual acceleration in roast energy. Samples were collected every minute from the roaster to measure the TA. The second objective was to assess how the TA dynamics depended on the origin and post-harvesting method used to process the coffee beans, examining a smaller subset of roast profiles. An important finding is that the dynamics of TA development depend sensitively on the roast profile, but key features like the peak TA are conserved regardless of roast profile or bean origin.

## Materials and methods

### Overview of the experimental design

**Figure 1:**
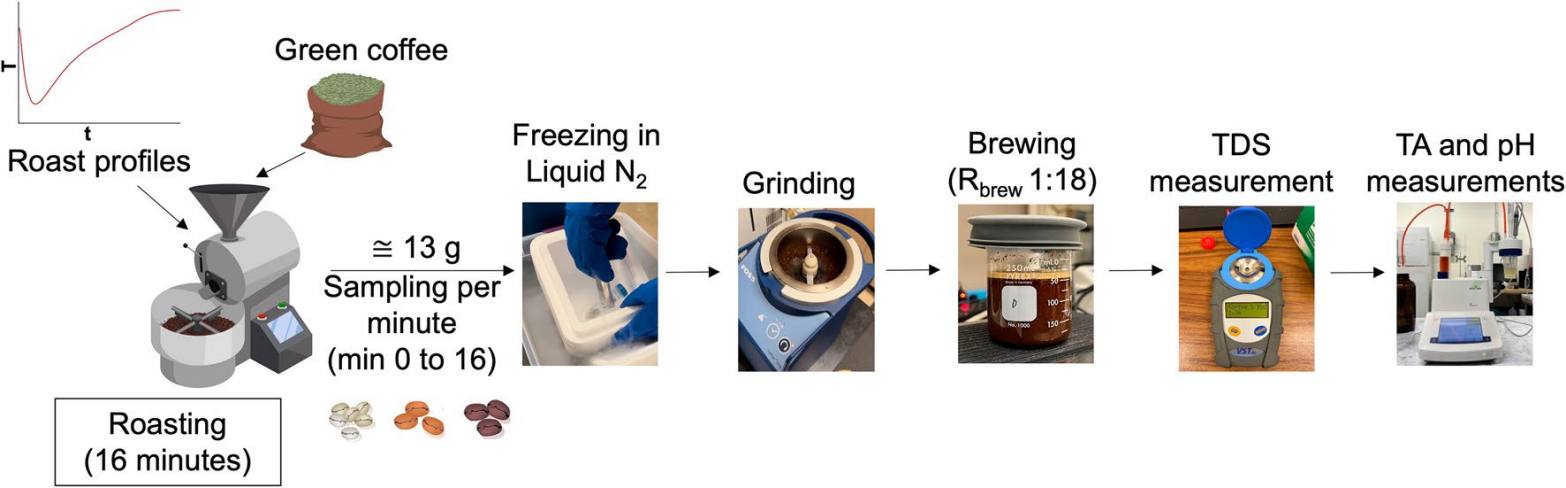
Schematic representation of the experimental procedure for roasting, sample collection, and analysis.

This study was conducted at the Coffee Center at the University of California, Davis from July to December 2022. To assess the relationship between roast profile and TA development, all roasts were performed in a 5-kg batch roaster (P5 model 2, Probat GmbH, Emmerich am Rhein, Germany) powered by natural gas. Our first set of experiments examined an African washed arabica coffee. The seven roast profiles were denoted as “fast start” (FS), “slow start” (SS), “medium” (MD), “production” (PR), “exaggerated flick” (EF), “negative rate of rise” (NRoR), and “extended Maillard” (EM), each of which is described in more detail below. Each roast lasted 16 min, during which samples were collected at one-minute intervals, yielding 17 total samples per roast (16 samples during the roast plus its corresponding green coffee sample). For the second objective, we investigated a smaller subset of roast profiles (FS, SS, and EM) using two additional coffees: a Central American honey-processed coffee and an Indonesian washed coffee, chosen for their very different taste profiles. Each roast was performed in triplicate, where we took care to modulate the energy inputs to the roaster to achieve a similar roast profile within all three replicates. Thus, we performed a total of 39 experimental roasts (7 × 3 for objective one and 2 × 3 × 3 for objective two), with 17 samples per roast, yielding a total of 663 samples. All samples were brewed to equilibrium using full immersion and then analyzed for their TA and pH levels. Figure 1 provides an overview of the experimental procedure.

### Green coffee

Green coffee (*Coffea arabica*) beans from three different origins (geographical locations) and postharvest processes were used: an African washed coffee from Uganda Sipi Falls (USF), a Central American honey processed coffee from El Salvador Ataco (ELS), and an Indonesian washed coffee from Sumatra (SUM). The USF and SUM coffees were donated by Royal Coffee Inc. (Oakland, CA, USA), and the ELS coffee was donated by Bridge Coffee Co. (Marysville, CA, USA). Green coffee beans were commercially purchased by these importers, which comply with institutional, national, and international guidelines and legislation. Thus, permits and licenses to procure the samples are not required. After receiving them, the green coffees were re-packed and labeled into smaller 1 kg jute sacks (Model No. S-8423, Uline, Pleasant Prairie, WI, USA), fastened with a zip tie, and stored in an environmental chamber (Caron Inc., model 7000–25, Marietta, OH, USA), with conditions set to mimic typical industry warehouse storage conditions of 25 °C and 60% relative humidity. After a minimum 10-day storage period, the coffee beans reached a 10.5 ± 0.5% wet basis moisture content, as determined by the method outlined in Anokye-Bempah et al.^23^.

### Roasting equipment and roast profiles

A natural gas Probat P5 drum coffee roaster with a 5-kg capacity was used in this study. Before each roast, the roaster was preheated to 210 ± 5 °C for at least 30 min to stabilize the drum temperature. To properly monitor changes in temperature within the roaster drum and to allow consistent airflow through the roast, we only loaded 4 kg of green coffee into the roaster, representing 80% of the roaster’s capacity. Roasting parameters such as roast temperature, roast time, and the rate of temperature increase per 30 s, commonly known as the rate of rise (RoR), were monitored, and recorded using Cropster Roasting Software (version 4.15.2, Cropster Inc, Sacramento, CA, USA, 2022).

**Figure 2:**
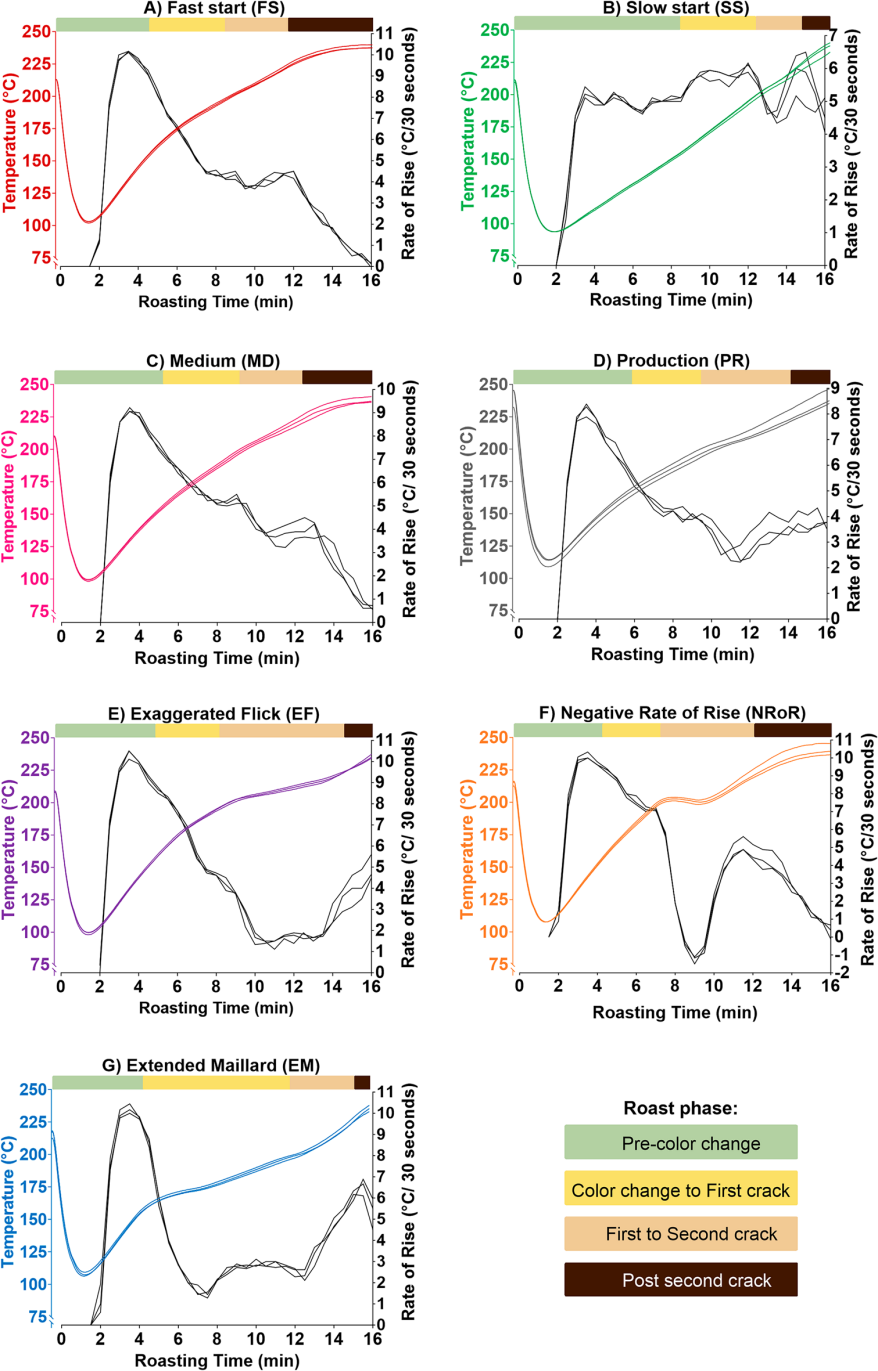
Roast profiles used to roast the African washed coffee. The colored lines in each subfigure represent temperature vs. time in the roaster drum, with each line depicting one of three replicates per roast profile. The black lines indicate the corresponding RoR curve for each profile. The colored rectangle above each subfigure denotes the roast phases.

**Figure 3:**
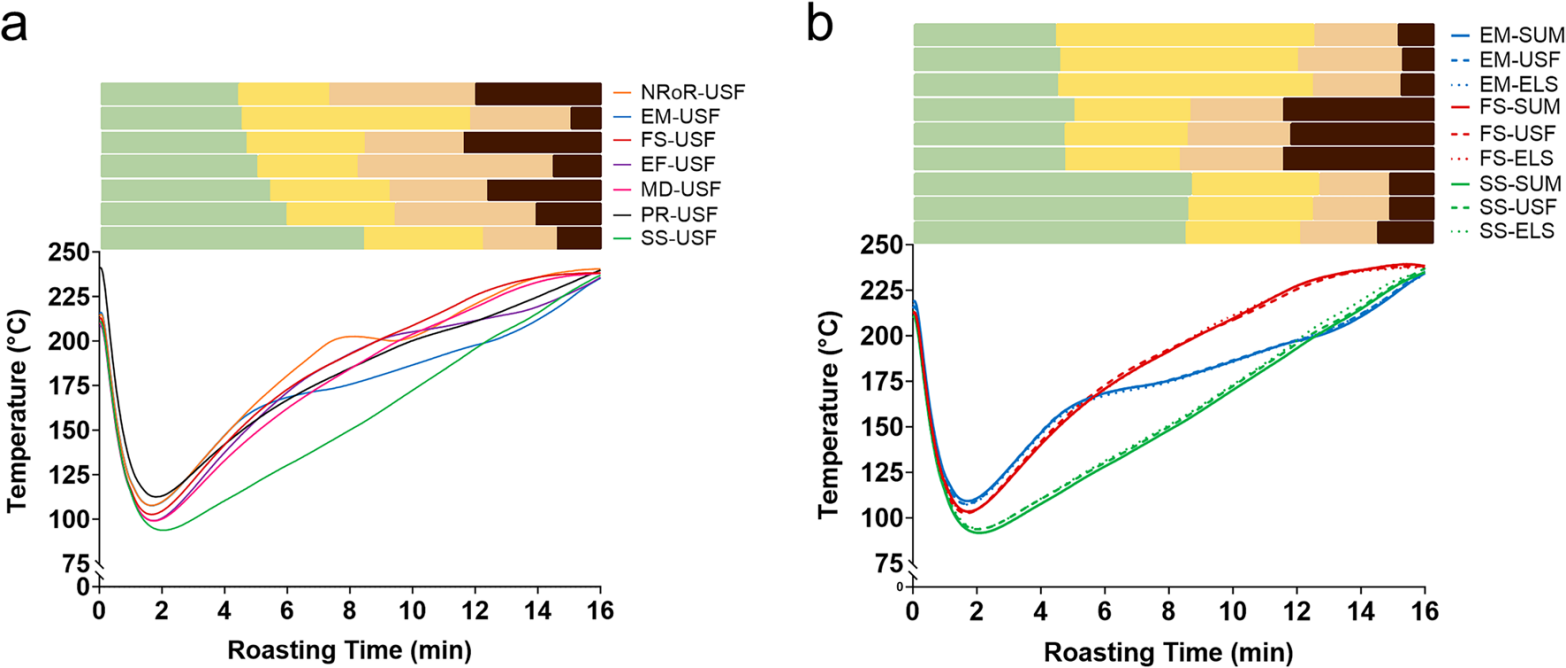
(**a**) Direct comparison of the roast profiles (NRoR, EM, FS, EF, MD, PR, SS) used to roast the African washed (USF) coffee. (**b**) Roast profiles used to roast the three different green coffees; African washed, Central American honey processed (ELS), and Indonesian washed coffee (SUM). Colored lines represent temperature vs. time in the roast drum, with each line depicting the mean of three replicates per roast profile.

The seven roast profiles that were explored in this study, including FS, SS, MD, PR, EF, NRoR, and EM, represent a variety of typical coffee industry roast profiles. Differences in the profiles were achieved by varying the energy dynamics and heat intensity of the roaster throughout the roasting process, by modulating the natural gas flow and airflow. All roasts were maintained at a similar starting and final temperature of 215 ± 8 °C and 237 ± 2 °C, respectively, and lasted a total of 16 min to allow sufficient time to investigate subtle changes in the TA from the green coffee stage to the burnt/charred coffee stage. Qualitative descriptions of each roast profile are outlined below in “Fast start (FS)” to “Extended maillard (EM)” sections, while quantitative measurements of the overall changes in the roast profiles are shown in Table 1. For visual representation, Fig. 2 shows the seven roast profiles used to roast the African washed coffee in objective one, including their respective RoR curves, while Fig. 3a displays a summary of these roast profiles. Subsequent analysis of the collected roast profile data revealed that the FS, SS, and EM profiles were the most distinct roast profiles (see supplementary Table S1). Thus, we selected these three specific profiles to roast the Indonesian washed and Central American honey processed green coffees. Figure 3b summarizes the three roast profiles used to roast the Indonesian washed and Central American honey processed coffees in objective two. Individual plots of the roast profiles used in objective two and their respective RoR curves can be found in the supplementary Figure S1. The order of each roasting experiment was randomized, and each roast was repeated three times. Each roast had four distinguishable phases. (1) The “pre-color change” phase corresponds to the time between the beginning of the roast and time at which the beans have visibly altered in color from the initial greenish yellow to yellowish-brown. This phase is often referred to as the drying phase, since it is believed the majority of water vapor loss occurs during this phase. (2) The second phase is denoted as “color change to first crack”, corresponding to the time between color change and when the first popping sounds are heard (first crack). This phase is commonly referred to as the Maillard phase since many roasters associate the browning with Maillard reactions^24^, although Maillard reactions are known to continue after first crack as well^25^. (3) Next is the “first to second crack” phase, corresponding to the time between first crack and when the second round of popping sounds are perceived (second crack). This phase is commonly referred to as the “development” phase. (4) Finally, we denote the “post second crack” phase as the time from the end of second crack to when the beans are expelled to the cooling tray. To our knowledge there is no common name for this phase, which typically involves charring and blackening of the beans and much smoke production. We emphasize that the duration of each of these phases varies depending on the roast profile.

**Table 1:** Roast profile parameters collected during roasting, including the initial and final rate of rise (RoR), as well as the duration and mean RoR for each roast phase.

Coffee type	Roast profile	Initial RoR (°C/30 s)	Final RoR (°C/30 s)	Roast Phase							
				Pre-color change		Color change to first crack		First to second crack		Post second crack	
				Duration (min)	Mean RoR (°C/30 s)	Duration (min)	MeanRoR (°C/30 s)	Duration (min)	MeanRoR (°C/30 s)	Duration (min)	MeanRoR (°C/30 s)
African washed	FS	10.15	0.08	5.0	7.97	3.5	5.39	3.5	4.39	4.0	1.58
	SS	5.26	4.56	8.5	5.07	4.0	5.79	3.5	5.2	1.0	4.83
	MD	9.11	0.63	5.5	7.92	4.0	5.51	3.0	3.89	3.5	2.14
	PR	8.19	3.69	5.0	7.09	4.5	4.56	4.5	3.13	2.0	3.75
	EF	10.37	4.93	5.0	7.86	3.5	5.97	6.0	2.13	1.5	4.32
	NRoR	10.11	0.32	4.5	7.98	2.5	7.72	5.0	2.58	4.0	2.46
	EM	10.20	5.32	4.5	7.88	7.5	2.80	2.0	3.60	2.0	5.83
Central American honey processed	FS	9.91	0.37	5.0	7.56	3.5	5.68	3.0	4.40	4.5	1.62
	SS	5.33	1.89	8.5	4.76	3.5	5.90	2.5	5.52	1.5	3.29
	EM	10.01	4.87	4.5	7.67	7.5	2.90	3.0	4.23	1.0	5.32
Indonesian washed	FS	9.96	0.01	5.0	8.76	4.0	5.45	2.5	4.46	4.5	1.68
	SS	5.07	3.72	8.5	4.72	4.0	5.72	2.5	5.49	1.0	4.23
	EM	9.90	5.43	4.5	7.51	7.5	2.79	3.0	4.18	1.0	6.02

The initial RoR reflects the highest positive RoR immediately after the turning point, or when the coffee beans and the roaster temperature equilibrate, on each roast curve. The final RoR indicates the RoR during the last minute of the roast.

#### Fast start (FS)

The FS roast profile is characterized by a high initial heat application, followed by a constant deceleration in roast energy (Fig. 2A). It has a relatively short pre-color change phase (4 min), with first crack occurring at approximately 8 min into the roast (Table 1).

#### Slow start (SS)

This profile is characterized by a low initial heat application, followed by a constant acceleration in roast energy (Fig. 2B). Compared to the FS profile, the SS roast profile has a longer pre-color change phase of approximately 8 min, with first crack occurring at 12 min into the roast (Table 1).

#### Medium (MD)

The characteristics of this roast profile fall between the FS and SS profiles (Fig. 2C, Table 1).

#### Production (PR)

This profile is achieved by maintaining the same roast energy setting throughout the roast (Fig. 2D, Table 1), i.e., without making any adjustments to the energy input. It is an easily reproducible profile and is therefore typically used for large-scale coffee roasting operations that feature multiple repeated roasts.

#### Exaggerated flick (EF)

A “flick” is a sudden increase in the RoR sometime after first crack, typically associated with the onset of exothermic reactions. This roast profile is considered by some roasters to be a common defect in coffee roasting that has a significant impact on the final flavor profile of the coffee^26^. Here we tested an “exaggerated” flick characterized by a very large increase in RoR around minute 13 (Fig. 2E, Table 1).

#### Negative rate of rise (NRoR)

This roast profile mimics an undesired loss of gas flow during roasting, particularly around the time of first crack (Fig. 2F). It is achieved by stopping the roast energy during first crack and subsequently increasing the roast energy to continue the roasting process (Table 1). The NRoR roast profile is believed by some roasters to seriously degrade final product quality^26^, although to our knowledge no published data supports this contention.

#### Extended maillard (EM)

This profile replicates a ‘baked’ roast profile by quickly moving through the pre-color change phase and extending the color change to first crack phase (Fig. 2G). It is thought to reduce sweetness and create “flat” or baked flavors in the final flavor profile of the coffee^26^. Of the seven roast profiles, the EM profile had the longest color change to first crack phase (Table 1).

### Roasting experiments and sampling procedure

During each 16-min roast, we collected a total of 17 coffee bean samples, with each sample weighing approximately 13 g, using the sample trier on the front of the drum. One green coffee sample was obtained before the roast, and 16 samples were collected at one-minute intervals throughout the roasting process. The collected samples were immediately weighed and divided into two separate 50-mL tubes (Falcon, Corning Inc., NY, USA): tube A, containing approximately 8 g of the sample, and tube B, containing approximately 5 g of the sample. The beans for tube B were first fan-cooled for sixty seconds using a standard square axial fan (Dayton, W. W. Grainger Inc., IL, USA) and room air, and then placed in the tube for subsequent moisture measurements not reported in this study. The tube A samples were immediately placed into the tube, and the entire tube was rapidly cooled in liquid nitrogen (N_2_). The tube was then temporarily stored in a cooler with dry ice to transport it to another lab for grinding. The beans from each tube A sample were ground in a water-cooled laboratory mill (KN 295 Knifetec™, FOSS Analytics, Hillerød, Denmark), and immediately stored in a –80 °C freezer until the day of brewing.

### Brewing

Samples were brewed using a standard full-immersion brew method, with an 18 to 1 water-to-ground coffee brew ratio^27^. Prior to brewing, the ground coffee samples were removed from the – 80 °C freezer and allowed to equilibrate to room temperature (25 °C) for 6 h. Immediately before brewing, purified water (pH 7.46) (Pure Life, Nestlé Waters North America, 2019) was heated to 94 °C using a Bonavita 1.7 L variable temperature electric kettle (Bonavita World, Woodinville, WA). Samples were then brewed by pouring 60 g of water in a circular motion into a 200 mL beaker containing 3.3 g of ground coffee to guarantee a uniform soaking of the coffee grounds. The brews were covered and kept on the laboratory bench for 30 min, as preliminary experiments indicated that this was the minimum required time for either green or roasted coffee samples to reach their maximum extraction, indicated by an observed plateau in the total dissolved solids (TDS) versus time^28^ (see supplementary Figure S2). During the extraction, brews were gently stirred with a spatula every 5 min to ensure adequate mixing. After extraction, coffee grounds were removed by pouring the brew through a Hario V60 dripper containing a Hario V60 filter paper (Hario, CA, USA). Each brew was collected in a clean 200 mL beaker. The TDS for each sample was immediately measured using a VST LAB Coffee III digital refractometer (VST Inc, Boston, MA, USA) to verify that maximum extraction was achieved.

### Titratable acidity (TA) measurements

TA measurements were performed immediately after brewing using an automatic titrator (G10s Titrator Compact, Mettler Toledo, USA), by titrating a 40 mL aliquot per brewed sample with 0.1 mol/L NaOH solution to an endpoint pH equal to 8.2 (a value chosen for consistency with prior measurements^2^). Therefore, throughout this study, TA is expressed as mL of NaOH per 40 mL sample. Initial brew pH was also measured by the pH meter in the titrator (see supplementary Figure S3 for pH results). The titrator pH meter was calibrated after every 17 measurements using acidic, neutral, and basic calibration standards (respectively pH 4.00 ± 0.01, color-coded red, pH 7.00 ± 0.01, color-coded yellow, and pH 10.00 ± 0.01, color-coded blue, all from VWR International, Radnor, PA, USA).

### Data and statistical analysis

Statistical analyses and data visualization were conducted using R version 4.2.3 (R Core Team, 2023). A statistical significance of α = 0.05 was used for all comparisons. For objective one, a two-way repeated measures analysis of variance (ANOVA) with TA as the repeated measure, time as the within-subjects factor, and roast profiles as the between-subjects factor, was used to determine if the roast profile and time significantly affect the TA. Additionally, to determine if TA significantly varied with time within the same roast profile, temporal TA values were compared using a one-way ANOVA. For objective two, a two-way repeated measures ANOVA was used to determine if the coffee origins significantly affect the TA. Whenever the ANOVA test was significant, differences were inferred by applying a post hoc Tukey Honest Significant Difference (HSD) test. Lastly, a one-way ANOVA was used to determine if there were significant differences between the maximum or peak TA values for all roast profiles and coffee origins.

## Results

### Effect of roast profiles on titratable acidity (TA)

**Figure 4:**
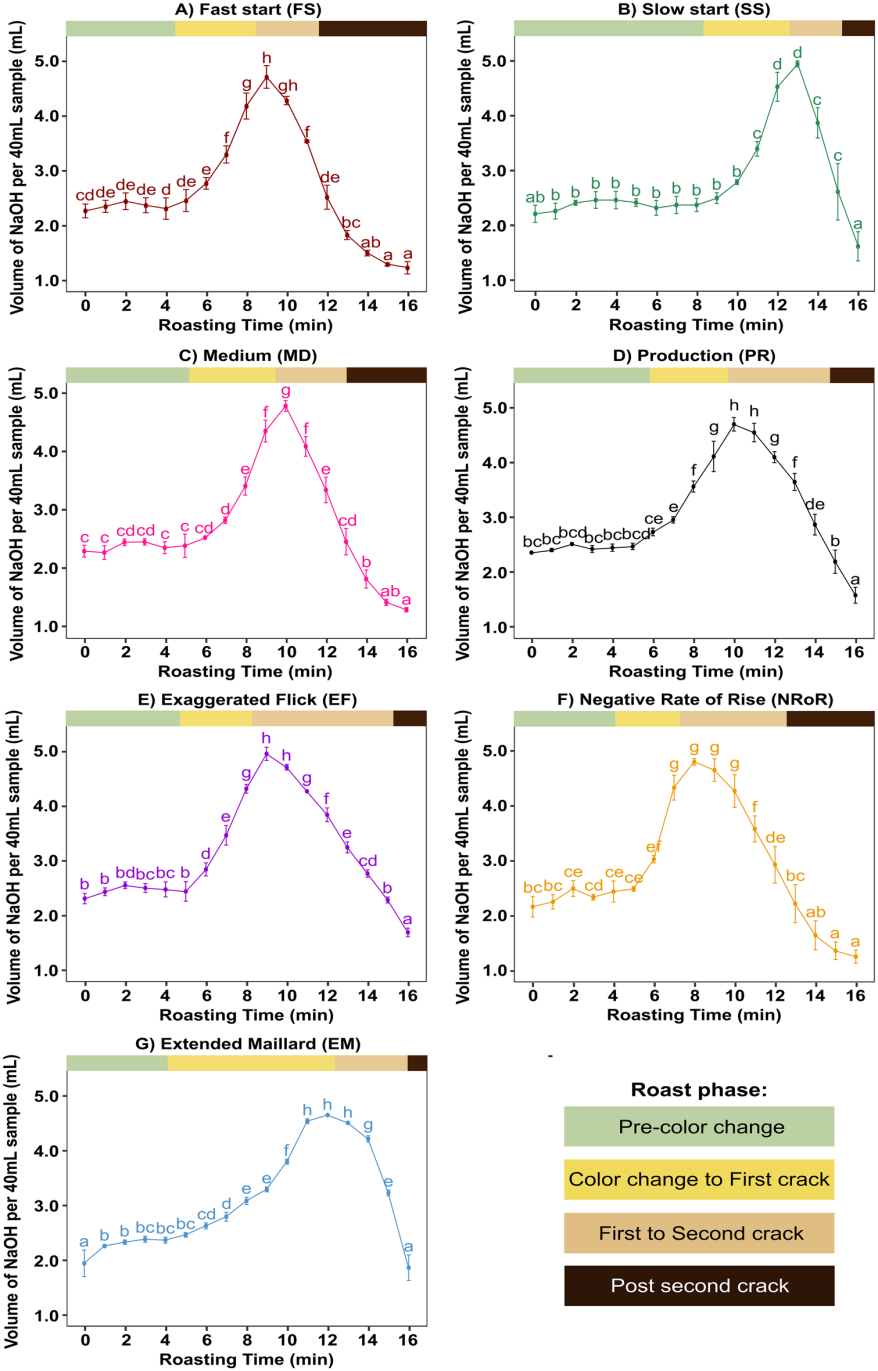
The TA of African washed coffee samples acquired with different roast profiles: fast start (FS), slow start (SS), medium (MD), production (PR), exaggerated flick (EF), negative rate of rise (NRoR), and extended Maillard (EM). The colored points in each subfigure represent the mean of three sample replicates and the error bars represent one standard deviation from the mean. Time points within a given roast profile that share the same lowercase letter(s) are not significantly different (p > 0.05).

Figure 4 shows the changes in TA among the seven roast profiles for the African washed coffee. The mean initial TA was 2.2 ± 0.3 mL NaOH/40 mL sample (pH = 5.9 ± 0.1), which reflects measurements across 7 × 3 = 21 sample replicates of the same green coffee. For all tested roast profiles, we observed a significant increase in TA from the beginning of the roast until the first to second crack phase where the TA peaked. The TA then significantly decreased until the end of the roast (Fig. 4). This general trend in the dynamics of TA during roasting qualitatively accords with previous studies^12^.

Our results, however, reveal two new observations. First, regardless of the roast profile, the peak TA always occurred concurrently during first crack, with an average peak TA of 4.9 ± 0.2 mL NaOH/40 mL sample (averaged over all roast profiles and coffee origins). For example, in the FS roast profile (Fig. 4a), the peak TA occurred during the tenth minute of the roast, coinciding within a few seconds of the onset of first crack. Similarly, for the SS profile, the peak TA occurred during the thirteenth minute of the roast, again corresponding to the start of first crack for that roast profile (Fig. 4b). This concurrence of peak TA and first crack was consistent across all tested roast profiles and coffee origins.

The second new observation is that regardless of roast profile, at the onset of second crack the TA had decreased to almost exactly the initial TA value. For instance, in the FS, EF, and PR roast profiles, the TA values at 2nd crack were 2.5 ± 0.2 mL NaOH/40 mL, 2.7 ± 0.1 mL NaOH/40 mL, and 2.8 ± 0.2 mL NaOH/40 mL respectively, similar to their initial TA value of 2.2 ± 0.3 mL NaOH/40 mL, as seen in Fig. 4.

**Figure 5:**
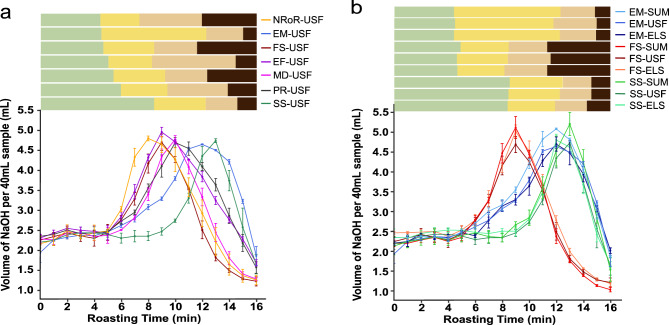
(**a**) Direct comparison of the TA during roasting of the African washed coffee (USF) using seven different roast profiles. Each colored line represents a different roast profile, and the error bars indicate one standard deviation from the mean within three roasting replicates (**b**) direct comparison of the TA for the three green coffees (USF, SUM, and ELS), roasted using fast start (FS), slow start (SS), and extended Maillard (EM) roast profiles.

While the overall trend in TA was consistent across roast profiles, the different roast profiles strongly affected the TA dynamics (Fig. 5a). For the FS roast profile, the changes in TA occurred at a faster rate as compared to the SS roast profile, with the peak TA occurring during the 8th minute of the roast. In contrast, for the SS roast profile, the peak TA occurred in the 13th minute of the roast (Fig. 4). Compared to the other roast profiles, the EM profile, which had the longest color change to first crack phase, exhibited a relatively slow increase in TA during the color change to first crack phase. Surprisingly, the NROR and EF roast profiles, which are believed to be associated with common roasting defects, such as flat or baked flavors in the final product quality, yielded TA profiles almost indistinguishable from the MD roast profile.

A two-way repeated measures ANOVA performed on the TA dynamics showed a significant main effect of roast profile (F (6, 12) = 20.4, p < 0.001), a significant main effect of time (F (16, 32) = 991.17, p < 0.001), and a significant interaction between roast profile and time (F (96, 192) = 58.95, p < 0.001), indicating that TA was significantly affected by both roast profile and roasting time. Post-hoc Tukey tests indicated that all seven roast profiles were significantly different from one another in their overall effect on the TA dynamics during roasting, with the FS, SS, and EM profiles being the most significantly different (bold *F* and *p* values in Supporting Information Table S1). In other words, although the trends of peak TA at first crack and original TA at second crack were conserved across all roast profiles, the specific dynamics of when those TAs were achieved was highly impacted by the roast profile.

### Effect of green coffee origin/postharvest processing methods on TA

To test whether the trends shown in Figs. 4 and 5a were unique to that specific African washed coffee, we repeated the measurements with two other coffees. Figure 5b shows how the TA varied with roast profile for all three different green coffee origins. The overall trends for the Central American honey processed and Indonesian washed coffees are extremely similar to the African washed coffee analyzed in Fig. 4. The TA increases up to a peak value during first crack, followed by a decrease in TA towards the end of the roast (Fig. 5b). Furthermore, the ANOVA results showed no significant statistical differences in TA between the three origins and processing methods for the FS, SS, and EM roast profiles (p > 0.05). This result suggests that changes in TA during roasting are mainly governed by the roast profile, not the specific origin of the beans.

**Figure 6:**
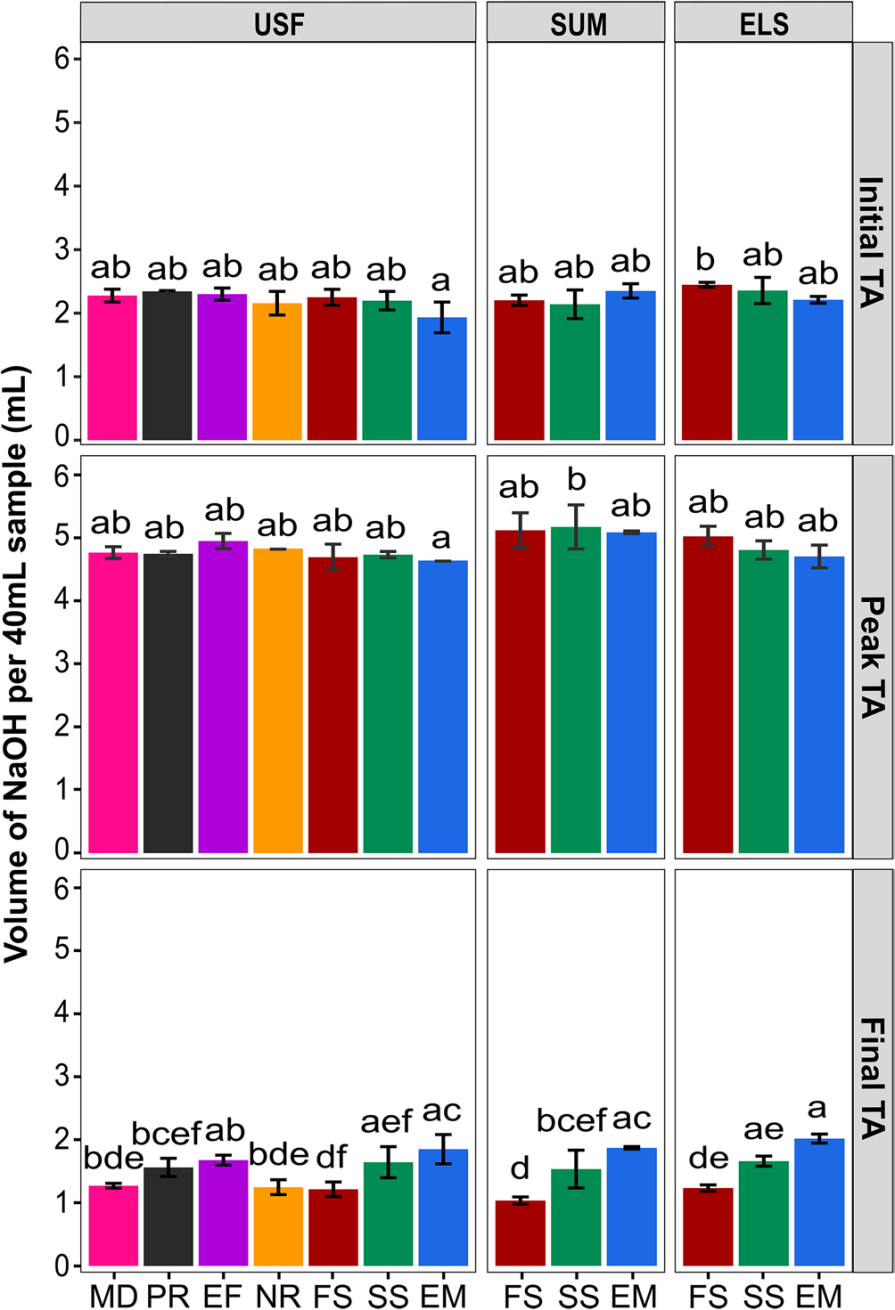
Initial TA, Peak TA, and Final TA for the seven roast profiles (MD, PR, NRoR, EM, FS, EF, SS) and three green coffees (USF, SUM, and ELS). Error bars represent the standard deviation from the mean of three roasting replicates. Bars with different lower-case letter(s) within the initial, peak, or final TA, indicate significant differences in TA (P < 0.05) through all of the origins, as determined by Tukey post-hoc test.

A surprising aspect of our results is that the peak TA appeared similar regardless of roast profile or origin. To assess this observation more quantitatively, we calculated the mean and standard deviation of each peak TA across the three replicate roasts (Fig. 6). Across all roast profiles and for all three coffee origins and processing methods the peak TA had an average of 4.9 ± 0.2 mL NaOH/40 mL sample. In other words, the peak TA did not depend on the roast profile or the coffee origin (at least for those tested). The average peak TA for the USF coffee was 4.8 ± 0.1 mL NaOH/40 mL sample, while the average peak TA for the ELS and SUM coffees were 4.9 ± 0.3 mL NaOH/40 mL sample and 5.1 ± 0.2 mL NaOH/40 mL sample respectively, representing only a corresponding 2% and 3% increase in average peak TA values compared to the USF coffee. A one-way ANOVA and post hoc Tukey test showed no significant differences in peak TA across all profiles and origins, except for the SS-SUM and EM-USF coffees (Fig. 6). The SS-SUM exhibited a higher peak TA of 5.2 ± 0.35 mL NaOH/40 mL sample, while the EM-USF had a lower peak TA of 4.6 ± 0.01 mL NaOH/40 mL sample compared to the average peak TA. In addition to the peak TA, Fig. 6 also shows the initial and final TA for all roast profiles and coffee origins. There were no significant differences in the initial TA for the roast profiles and coffee origins; likewise, there were no significant differences in the final TA for different coffee origins. However, the ANOVA showed significant differences in the final TA among the roast profiles. The FS, NROR, and MD roast profiles had low final TA values with an average of 1.2 ± 0.1 mL NaOH/40 mL sample, while the EM, PR, SS, and EF roast profiles had relatively high final TA values with an average of 1.7 ± 0.2 mL NaOH/40 mL sample. Despite the significant differences, all final TAs were within a factor of 2 mL NaOH/40 mL sample and had a minimum of 1 mL NaOH/40 mL sample.

## Discussion

The goal of this study was to investigate how roast profiles affect the TA of coffee during roasting, using green coffee from three different origins. Overall, this study yielded several important findings. First, we found that roast profiles significantly affect the dynamics of TA during coffee roasting. Previous studies^12,21,22^ have shown significant differences in TA for coffee roasted with isothermal roast profiles in laboratory-scale roasting. However, the use of a commercial-scale roaster along with a diverse range of industry-standard roast profiles in our study, strongly corroborate these general trends for commercial-scale roasting applications. Our results suggest that by applying specific and different roast profiles, roasters can control the TA of coffee during roasting, and thus regulate its perceived acidity or sourness. For instance, a nine-minute roast using the SS roast profile will produce coffee with a TA of 2.4 mL NAOH/40 mL sample, while a nine-minute roast using an EF roast profile will produce coffee with twice the amount of TA, as illustrated in Fig. 5a.

Secondly, we observed that regardless of the roast profile or coffee origin, the peak TA always occurred during first crack, as seen in Figs. 4 and 5. This finding is consistent with previous studies that also reported that maximum TA occurs near the end of first crack during laboratory-scale roasting^12,22^. A third key finding was that the peak TA exhibited almost no significant differences among roast profiles and coffee origins. In other words, all seven distinct roast profiles and coffee origins had similar peak TA values with an average of 4.9 ± 0.2 mL NaOH/40 mL. As shown in Fig. 6, only the SS-SUM and EM-USF coffees were significantly different (p-value = 0.0027). These results suggest that the peak TA does not depend on the roast profile but may depend on other factors such as the inherent composition of the coffee beans. The Peak TA values recorded in this study are in agreement with previous studies, including Wang and Lim^12^ who reported maximum TA values of 5.2 mL NaOH/40 mL sample. Another notable observation is that the TA decreased to its initial value by the 2nd crack, across all roast profiles and origins (Figs. 4 and 5). Although previous studies have reported a decrease in TA toward the end of roasting^12,14,18,19^, no information on the decline of TA to its initial value by 2nd crack has been reported in the literature.

It is well established that the TA of coffee generally increases up to a peak value during roasting, then decreases as the roast progresses^4,15^. Although we did not measure any specific acid concentrations in this study, prior work provides some insight on possible interpretations of the observed trends in TA^4,18^. The increase in TA is likely attributed to the formation of formic and acetic acids from carbohydrates, while the decrease in TA results from the degradation of organic acids, such as citric and malic into succinic, fumaric, and maleic acids. It is remarkable that despite the complicated network of chemical reactions that presumably have different temperature dependencies, the peak TA is insensitive to how quickly those temperatures are reached, as evident in this study.

As observed in Fig. 5b, TA did not vary significantly between the different coffee origins or postharvest processing methods in contrast to previous studies that reported significant differences in TA during roasting from different green coffees^22^. A potential reason is that the three green coffees included here contained similar initial TA as shown in Fig. 6. Future work should compare green coffees with significantly different TA values to ascertain if this will significantly impact the TA levels and dynamics during roasting.

## Conclusion

Overall, our study yielded four key results. First, we found that industry-standard roast profiles affect the TA of coffee during commercial-scale roasting. Second, we discovered that the peak or maximum TA always occurred during first crack regardless of the roast profile, and the peak TA values are similar across all roast profiles and coffee origins. Third, we observed that TA decreased to its initial value by second crack for all roast profiles. Lastly, we reported that different origins and processing methods do not significantly affect TA during roasting, at least for the samples tested here. Our results expand the limited existing academic literature on the behavior of TA during commercial-scale roasting and provide detailed data that provides insight on how roast profiles can be manipulated to achieve desired sourness in coffee. We emphasize here that we performed only simple chemical measurements of TA. Since TA represents the total concentration of all acids present, it is still unclear how the individual acids in coffee are affected by roast profiles during roasting. More detailed chemical analysis of specific acid concentrations versus roast profile would provide more insights into acidity development during roasting. Future work should also evaluate the sensory quality, specifically the acidity or perceived sourness of coffee roasted, with different roast profiles to assess the effects of varied TA profiles on the crucial sensory aspect of coffee. Finally, we note that many coffee industry practitioners are not equipped to readily measure TA, but do have means of measuring the color of the roasted coffee. Future work should examine correlations between the color of the roasted coffee and the TA and corresponding sensory qualities.

### Supplementary Information

Supplementary Information.

## Data Availability

All relevant data are available from the corresponding author upon request.
