# Supplementary material for: The effect of roast profiles on the dynamics of titratable acidity during coffee roasting
Source: Sci Rep. 2024 Apr 8;14:8237. doi: 10.1038/s41598-024-57256-y (PMC11002029; doi:10.1038/s41598-024-57256-y)
Supplement: Supplementary file 1 — Supplementary Information. [file 41598_2024_57256_MOESM1_ESM.pdf]

Supplementary Information:

How Roast Profile Affects the Dynamics of Titratable Acidity during Coffee Roasting

Laudia Anokye-Bempah, Timothy Styczynski, Natalia Andrade Teixeira Fernandes, Jacquelyn Gervay-Hague, William D. Ristenpart, & Irwin R. Donis-González

Fig. S1. Roast profiles for Central American honey processed and Indonesian washed coffees.

Fig. S2. Total Dissolved Solids (TDS) vs. brew extraction time.

Fig. S3. Changes in pH during coffee roasting.

Table S1. One-way ANOVA results for roast profiles.

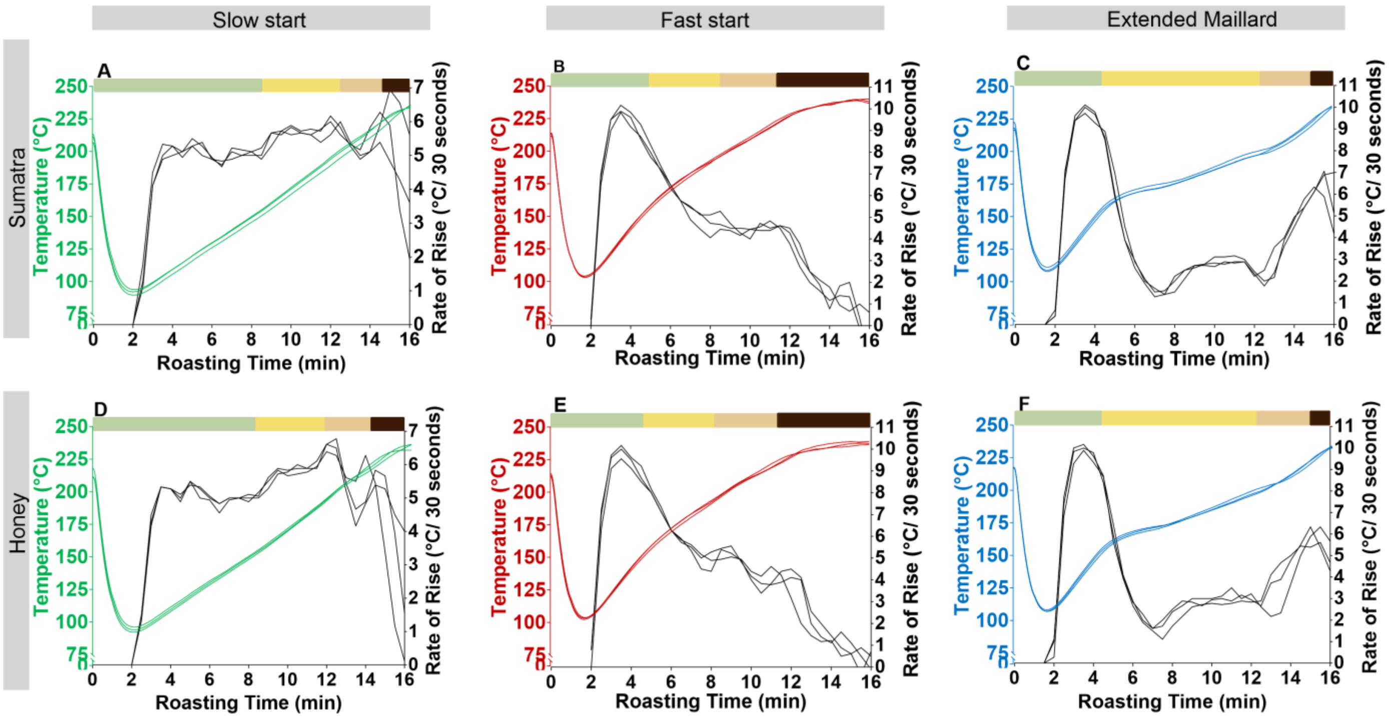

Supplementary Figure S1. Roast profiles used to roast the Central American honey processed (ELS) and Indonesian washed coffee (SUM). A) Slow start- SUM B) Fast start-SUM, C) Extended Maillard-SUM, D) Slow start- ELS, E) Fast start-ELS, F) Extended Maillard- ELS. Colored lines in each subfigure represent temperature vs time in the roast drum, with each line depicting one of three replicates per roast profile. Black lines indicate the corresponding RoR curve for each profile. The colored rectangle above each subfigure shows the roast phases for the specific roast profile.

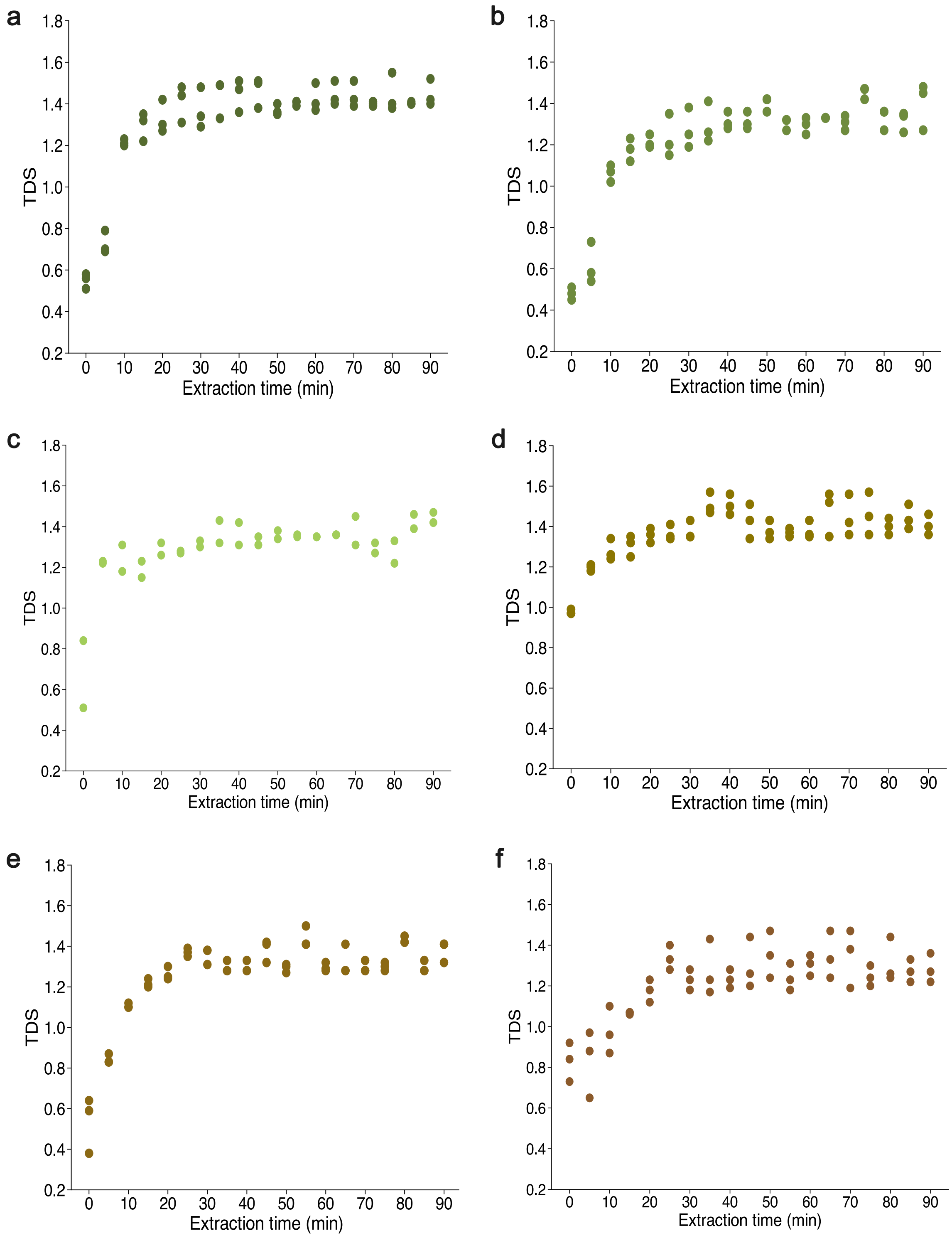

Supplementary Figure S2. Scatter plots of Total Dissolved Solids (TDS) vs. brew extraction time of six different coffee samples differentiated by the sample's roasting time: a) green coffee (roast time 0), b) 1 minute roast time, c) 2 minutes roast time, d) 3 minutes roast time, e) 4 minutes roast time, f) 5 minutes roast time. Colored points in each subfigure represent the relative coffee bean color at the specific roast time, with each point depicting one of three replicates per TDS measurement.

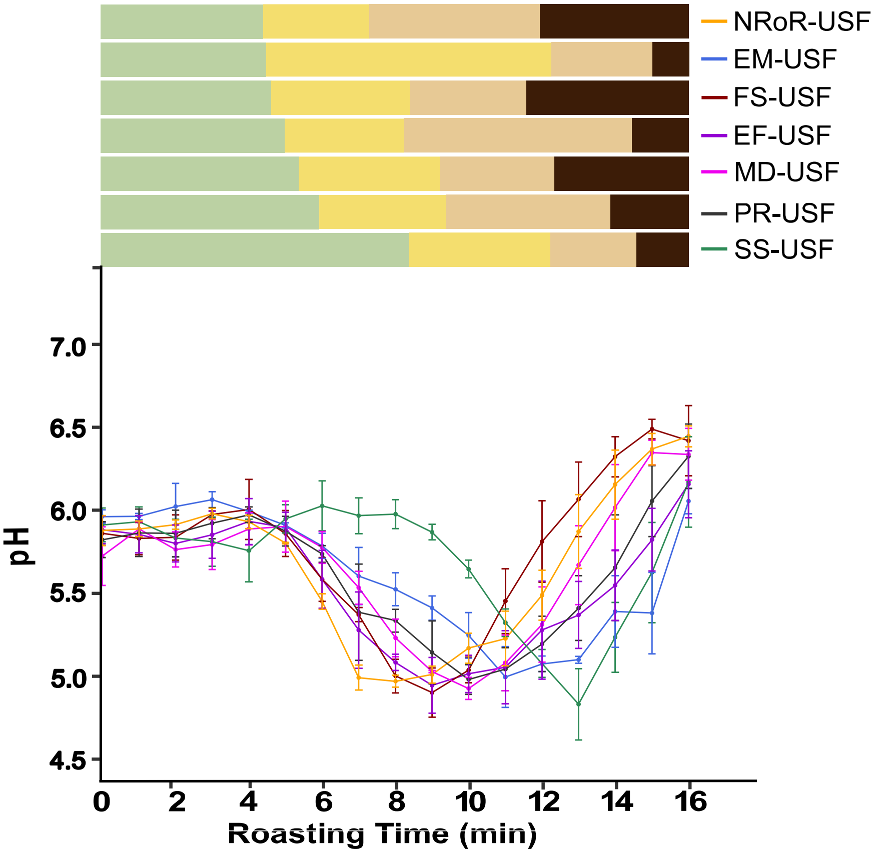

Supplementary Figure S3. Changes in pH during roasting of the USF coffee using seven different roast profiles. Each colored line represents a different roast profile, and the error bars indicate one standard deviation from the mean within three roasting replicates.

Supplementary Table S1: Table of F-ratios from one-way ANOVA with corresponding F-ratio and p-values. Bold values represent the most distinct roast profiles among the seven considered.

Roast Profile	F-ratio	P-value
**Fast Start**	**26293.27**	**3.07×10^{-61}**
Medium	6510.15	1.53×10^{-51}
Exaggerated Flick	5754.16	1.10×10^{-50}
**Slow start**	**5253.114**	**4.74×10^{-50}**
**Extended Maillard**	**4597.74**	**3.99×10^{-49}**
Negative rate of rise	1711.975	2.87×10^{-42}
Production	1373.84	9.65×10^{-41}
